# Role of Mixed-Species Stands in Attenuating the Vulnerability of Boreal Forests to Climate Change and Insect Epidemics

**DOI:** 10.3389/fpls.2021.658880

**Published:** 2021-04-29

**Authors:** Raphaël D. Chavardès, Fabio Gennaretti, Pierre Grondin, Xavier Cavard, Hubert Morin, Yves Bergeron

**Affiliations:** ^1^Institut de Recherche sur les Forêts, Université du Québec en Abitibi-Témiscamingue, Rouyn-Noranda, QC, Canada; ^2^Groupe de Recherche en Écologie de la MRC-Abitibi, Université du Québec en Abitibi-Témiscamingue, Amos, QC, Canada; ^3^Direction de la Recherche Forestière, Ministère des Forêts, de la Faune et des Parcs, Québec, QC, Canada; ^4^Département des Sciences Fondamentales, Université du Québec à Chicoutimi, Saguenay, QC, Canada; ^5^Université du Québec à Montréal, Montréal, QC, Canada

**Keywords:** Forest tent caterpillar (*Malacosoma disstria*), spruce budworm [Choristoneura fumiferana (Clem.)], summer heat stress, growing season length, climate-growth relations, trembling aspen (*Populus tremuloides* Michx.), *Picea mariana* (Mill) B.S.P, stand mixture

## Abstract

We investigated whether stand species mixture can attenuate the vulnerability of eastern Canada’s boreal forests to climate change and insect epidemics. For this, we focused on two dominant boreal species, black spruce [*Picea mariana* (Mill.) BSP] and trembling aspen (*Populus tremuloides* Michx.), in stands dominated by black spruce or trembling aspen (“pure stands”), and mixed stands (M) composed of both species within a 36 km^2^ study area in the Nord-du-Québec region. For each species in each stand composition type, we tested climate-growth relations and assessed the impacts on growth by recorded insect epidemics of a black spruce defoliator, the spruce budworm (SBW) [*Choristoneura fumiferana* (Clem.)], and a trembling aspen defoliator, the forest tent caterpillar (FTC; *Malacosoma disstria* Hübn.). We implemented linear models in a Bayesian framework to explain baseline and long-term trends in tree growth for each species according to stand composition type and to differentiate the influences of climate and insect epidemics on tree growth. Overall, we found climate vulnerability was lower for black spruce in mixed stands than in pure stands, while trembling aspen was less sensitive to climate than spruce, and aspen did not present differences in responses based on stand mixture. We did not find any reduction of vulnerability for mixed stands to insect epidemics in the host species, but the non-host species in mixed stands could respond positively to epidemics affecting the host species, thus contributing to stabilize ecosystem-scale growth over time. Our findings partially support boreal forest management strategies including stand species mixture to foster forests that are resilient to climate change and insect epidemics.

## Introduction

The boreal forest is the second largest biome on Earth, providing humans with ecosystem services that include sustainably harvested wood, carbon storage, and freshwater resources (Gauthier et al., 2015). Yet, there are growing concerns for the boreal forest about climate change affecting ecosystem services through direct and indirect impacts on stand dynamics and disturbances (Seidl et al., 2016). To address these concerns, landscape managers are encouraged to follow a suite of strategies to minimize vulnerabilities to climate change and reduce potential losses in stand productivity and commercial species abundance (Gauthier et al., 2014). Among the suggested strategies is the promotion of tree species mixture within stands, i.e., mixed-species stands (Felton et al., 2016).

The role of species mixture was investigated in reviews that compile a suite of findings identifying the beneficial effects of mixed-species stands over single-species stands including increased stand-level biodiversity and productivity (e.g., carbon sequestration, nutrient cycling, and water-use efficiency), decreased risk of damage caused by some disturbances (e.g., pathogens, pests, and windthrow), and diversified forestry production over time (Lilles and Coates, 2013; Felton et al., 2016; Liu et al., 2018). However, these reviews also mention challenges and disadvantages for the promotion of mixed-species stands relative to single-species stands. Specifically, the beneficial effects of species mixture may be confounded in research studies with the influences of site type, density, and tree age (Lilles and Coates, 2013). Species mixture may also bring some disadvantages, such as increased risks to browsing, higher logging costs, and less management simplicity (Felton et al., 2016). More importantly, studies that focus on understanding the dynamics in mixed-species stands and how these compare with single-species stands remain few, and thus limit our assessments of the benefits and disadvantages of these stands with respect to climate change and its impacts (Liu et al., 2018).

Species mixture interacts with the effects of climate change, which is influencing the growth of trees in boreal forests of Canada in several direct and indirect ways. For example, higher temperatures promote longer growing seasons and shoot elongation (Bronson et al., 2009); whereas increased summer heat stress and reductions in water availability result in growth suppressions and physiological stresses (Deslauriers et al., 2014; Girardin et al., 2016a,b; Puchi et al., 2020). Yet, while many studies investigated how climate may directly affect the growth of boreal species (e.g., Jarvis and Linder, 2000; Girardin et al., 2008; Price et al., 2013; Girardin et al., 2014; Chen et al., 2016; D’Orangeville et al., 2016, 2018a,b; Hember et al., 2017; Chaste et al., 2019), these rarely assess given species’ growth responses to climate as a function of whether a stand is dominated by a single species or a mixture of species (hereafter, “pure stands” and “mixed stands,” respectively). Apart from direct influences on tree growth, climate change can also indirectly influence the growth of trees by accelerating and magnifying disturbances such as insect epidemics that increased in frequency and severity in some boreal forests of Canada (e.g., Chen et al., 2018; Navarro et al., 2018). Insect development, survival, fecundity, and dispersion, as well as host-tree susceptibility *via* drought for example can all be influenced by climate change (Jactel et al., 2019). Nonetheless, only few studies investigated how insect epidemics affect pure vs. mixed stands.

In our study, the objective was to assess how climate and insect epidemics impact the growth of given boreal tree species in mixed and pure stands of eastern Canada. We focused on two regionally dominant tree species, black spruce [*Picea mariana* (Mill.) Britton, Sterns, & Poggenburg] and trembling aspen (*Populus tremuloides* Michx.), in mixed stands and stands dominated by one species [i.e., pure black spruce stands (PBS) and pure trembling aspen stands (PTA), respectively]. We compared stand attributes, developed species, and stand specific basal area increment (BAI) chronologies to test climate-growth relations, and compared tree growth during epidemics of a black spruce defoliator, the spruce budworm (SBW; *Choristoneura fumiferana* Clemens), and a trembling aspen defoliator, the forest tent caterpillar (FTC; *Malacosoma disstria* Hübn.). Although generally SBW has been associated with low mortality of black spruce relative to other species, studies suggest it could become a more privileged species of the spruce budworm diet in the future (Lavoie et al., 2019). Furthermore, we developed a model to jointly evaluate the influence of climate attributes, namely growing season length and summer heat stress, and insect epidemics, on the growth of a given tree-species and stand composition type. We hypothesized that black spruce and trembling aspen in mixed stands have greater average growth than in pure stands, and that these species are less vulnerable to climate change and insect epidemics in mixed- relative to pure stands. Ultimately, our research provides information on the potential role of species mixture in attenuating the vulnerability of eastern Canadian to climate change and insect epidemics.

## Materials and Methods

### Study Area

**Figure 1:**
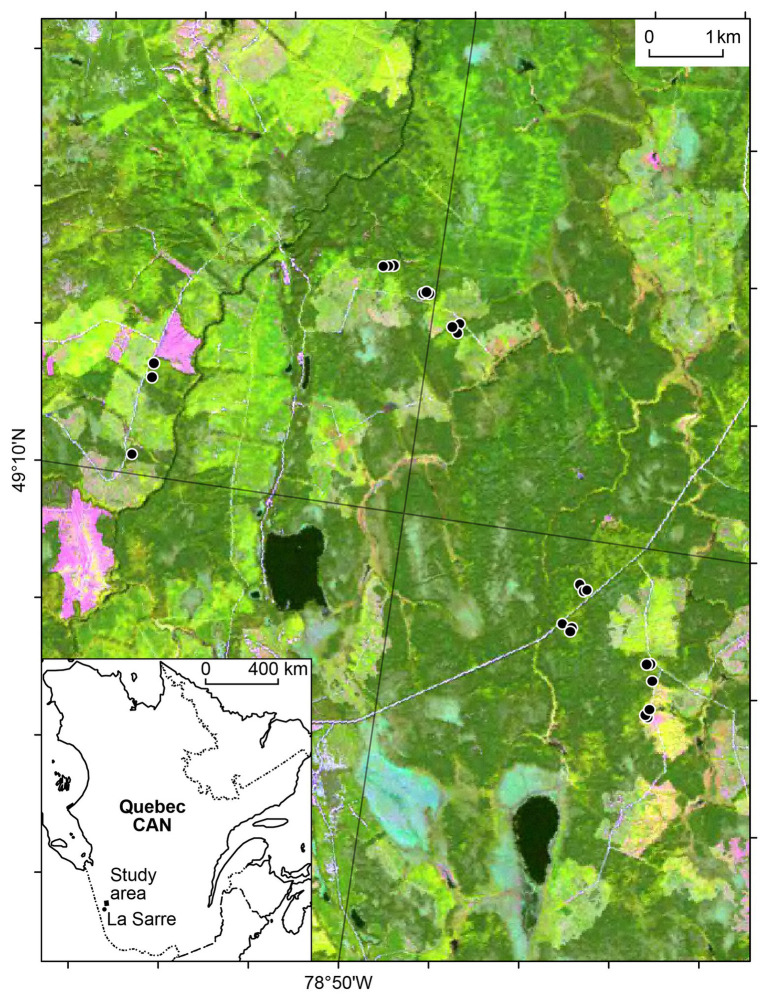
Map of the study area with the 24 sites (black dots) located in boreal forests of western Quebec, Canada (CAN). The inset map shows the study area relative to the La Sarre weather station.

The 36 km^2^ study area extends from 49°08'–49°11'N to 78°46'–78°53'W (Figure 1) in the clay belt of the black spruce-feather moss bioclimatic domain of western Quebec (Saucier et al., 2011). Though black spruce dominates in most forests of the study area, trembling aspen can also dominate in some stands or compose mixed stands with black spruce, particularly on clay surficial deposits (Ministère des Forêts, de la Faune et des Parcs, 2020a). Most stands within the study area originated from the same fire that occurred in 1916 (Légaré et al., 2005). The climate normals from 1971 to 2000 for the nearest weather station at La Sarre (48°47'N, 79°13'W, and 244 m.a.s.l., Environment Canada, 2020a) show mean annual air temperature is 0.7°C, with mean monthly temperatures of 16.9°C and −18.2°C for July and January, respectively. Total annual precipitation averages 889.8 mm, with 473.6 mm (53%) occurring as rain from May to September (Environment Canada, 2020a).

### Stand and Tree-Ring Data

We used stand and tree-ring data from Cavard et al. (2010). On similar clay deposits and drainage, Cavard et al. (2010) established 24 circular 400 m^2^ plots in eight blocks. At each plot, 20–40 trees were cored, and the diameter at breast height (DBH; 1.3 m above ground) and the species of each tree ≥5 cm DBH were recorded. In each block, three forest composition types were sampled: pure black spruce, pure trembling aspen, and mixed stands containing both species. Pure stands were defined as containing >75% of the dominant species in relative basal area, whereas mixed stands were mostly comprised of black spruce and trembling aspen but had no single species comprising more than 60% of the basal area (Cavard et al., 2010). For each core (*n* = 784), ring-width series were measured using a sliding-stage micrometer (Cavard et al., 2010).

We recovered the 784 ring-width series and reanalyzed them using CDendro version 9.0.1 (Larsson, 2017) and COFECHA to develop five chronologies with high inter-series correlations (>0.40) and using trees with pith or inner rings dating from before 1950. These five chronologies were for black spruce and trembling aspen in different composition types: (1) black spruce in PBS, (2) trembling aspen in PTA, (3) suppressed to intermediate black spruce in PTA, (4) trembling aspen in mixed stands, and (5) black spruce in mixed stands. Altogether, 197 ring-width series satisfied the chronology development criteria (*n* = 164 for black spruce and *n* = 33 for trembling aspen). For each series, we estimated the distance to pith (after Duncan, 1989) to calculate five chronologies of mean basal area increments (BAIs) using the R package “dplr” (Bunn, 2008). To exclude juvenile growth in the chronologies for following analyses over 1950–2005, we retained a minimum cambial age of 15 years.

To compare stand attributes across composition types, we assessed stand basal area and tree density, and basal area of individual trees according to their age. To test for significant differences across stand basal area and tree density, we conducted Kruskal-Wallis one-way ANOVA on ranks with *post hoc* Tukey tests. To assess significant differences in mean basal area according to tree age, we compared the uncertainty intervals defined by the SEM.

### Climate-Growth Relations

To test climate-growth relations, we required continuous monthly climate records. The nearest weather station at La Sarre had missing values between 1951 and 2004 (Environment Canada, 2020b). We therefore generated climate data, including monthly maximum, average, and minimum temperatures and monthly total precipitation from 1950 to 2005 for the study area with ClimateNA (Wang et al., 2016). Using the climate data and the mean BAI chronologies, we calculated correlations functions using the R package “treeclim” (Zang and Biondi, 2015). Specifically, we calculated Pearson’s Product moment correlations with their 95% CIs derived from 1,000 random bootstrapped samples (Efron and Tibshirani, 1993). In each analysis, we assessed a 17-month window from April of the previous year through September of the year of ring formation. We also assessed the early and late months of the growing season and the summer months by calculating mean values for April and September, and from June to August, respectively. This allowed us to examine the influences of growing season length and summer heat stress, which were identified in previous research as variables impacting tree growth in boreal forests near our study area (e.g., Hofgaard et al., 1999; Drobyshev et al., 2013), and resulted in significant correlations in our analyses. As the correlations in our analyses were weak to moderate during the previous year of ring formation, we only provide results for the year of ring formation.

### Influence of Specific Epidemic Years on Host and Non-host Species

We assessed if recorded SBW and FTC epidemic years within the region were associated to relevant growth anomalies for black spruce and trembling aspen in pure or mixed stands. Recorded epidemic years occurred in the 1970s for SBW (Ministère des Forêts, de la Faune et des Parcs, 2020b), and 1980, 2000, and 2001 for FTC (Bergeron et al., 2002; Ministère des Forêts, de la Faune et des Parcs, 2020c). We ranked mean BAI increases and decreases from 1 year to the next for the five chronologies between 1950 and 2005 (i.e., first-differenced chronologies), and we verified if the largest anomalies were simultaneous to epidemic years.

### Interactions Among Tree Growth, Species Mixture, Climate, and Insect Epidemics

We implemented linear models in a Bayesian framework using Markov Chain Monte Carlo (MCMC) sampling with Metropolis-Hastings steps (Gelman et al., 2014) to explain the mean BAI chronologies for each species and stand composition type as a function of climate and the occurrence of insect epidemics. The models simultaneously accounted for the impact of climate and epidemics to disentangle their effects. In the models, we tested the influence of (1) baseline mean BAI growth and mean BAI long-term linear trend for each chronology, (2) April and September average temperature anomalies as a proxy of growing season length, (3) June–August average temperature anomalies as a proxy of average summer heat stress, and (4) the presence of recorded SBW and FTC epidemics. We modeled predicted mean BAI for a specific year *t* and a specific chronology *y* as:

$$\begin{array}{l} BAI_{t,y}=\alpha \_Baseline_{y}+Trend_{t}\cdot \alpha \_Tr_{y}+SeasonLength_{t}\cdot \\ \;\;\;\;\;\;\;\;\;\;\;\;\;\;\;\;\;\;\;\;\;\;\;\;\alpha \_SL_{y}+SummerHeat_{t}\cdot \alpha \_SH_{y}+Budworm_{t}\cdot \\ \;\;\;\;\;\;\;\;\;\;\;\;\;\;\;\;\;\;\;\;\;\;\;\;\;\alpha \_SB_{y}+Caterpillar_{t}\cdot \alpha \_TC_{y} \end{array}$$

Where each *α* is an estimated parameter depicting the effect of a given factor on the mean BAI chronologies, *α*_*Baseline* represents the predicted baseline mean BAI of a chronology over the period 1950–2005 (units: cm^2^), *Trend* is an incremental number varying from −27 (1950) to +28 (2005) and thus *α*_*Tr* represents the coefficient of a long-term linear trend for a chronology linked to forest demography and dynamics (units: cm^2^/year), *SeasonLength* is the mean temperature anomaly for April and September (departures from the mean), *SummerHeat* is the June–August mean temperature anomaly (departures from the mean), *Budworm* is the SBW epidemic intensity, and *Caterpillar* is the FTC epidemic intensity. We used uniform priors for each *α*, and a Jeffreys prior for the SD of departures between the models and the observations (*σ*). Model posterior probability over iterations of the MCMC (60,000 iterations with the first 10,000 rejected) was then computed with the product between the model likelihood $\left(\Pi_{t=1950}^{2005}\;N\left(BAI_{t,y};\mu =SimBAI_{t,y},\;\;\sigma_{y}\right)\right)$ and priors of the hyperparameter vector $\left(p\left(\psi_{y}\right)\right)$.

The SBW epidemic intensity was identified as suggested by Rossi et al. (2018) with a 9-year triangular impact (0.2, 0.4, 0.6, 0.8, 1, 0.8, 0.6, 0.4, and 0.2) centered on 1974 (value = 1), the most widespread epidemic year during the 1970s in our study area (Ministère des Forêts, de la Faune et des Parcs, 2020b). The FTC epidemic intensity was assigned to 1 for the years 1980 and 2001 and to 0.5 for the year 2000 according to recorded epidemics (Bergeron et al., 2002; Ministère des Forêts, de la Faune et des Parcs, 2020c).

We plotted posterior distributions of the Bayesian model parameters (the six *α* for each model), including baseline mean BAI, mean BAI long-term linear trend, growing season length, summer heat, SBW impact, and FTC impact. We also implemented simpler models with subsets of the explanatory variables; however, we only present the full model because results were consistent among models. We considered that an overlap <10% across parameter posterior distributions for specific models was evidence of significant differences.

## Results

### Chronology and Stands Attributes

The five tree-ring chronologies had series intercorrelations ranging from 0.56 to 0.71, indicating robust crossdating and a similar response of trees within stands to climate and environmental variation (Table 1). Stand median densities were significantly higher in PBS than in PTA, whereas median basal areas were significantly larger in PTA than in PBS (Table 2). Mixed stands had average densities and basal areas. Based on cambial age, black spruce grew significantly faster in mixed stands than in pure black spruce or trembling aspen stands (Supplementary Figure 1). In PTA, black spruce was suppressed by aspen. For trembling aspen, the difference in growth according to cambial age was not significant, although aspen in pure stands revealed larger mean basal areas.

**Table 1:** Statistical characteristics of the black spruce and trembling aspen chronologies by stand composition type.

Species	Black spruce			Trembling aspen	
Composition type	Pure black spruce	Mixed	Pure trembling aspen	Pure trembling aspen	Mixed
No. of trees (radii)	61	73	30	22	11
Mean ring width (mm)	0.82	0.81	0.73	1.39	1.53
Mean sensitivity	0.16	0.21	0.22	0.32	0.27
SD (mm)	0.29	0.38	0.29	0.65	0.63
1st order autocorrelation	0.79	0.79	0.71	0.64	0.65
Series intercorrelation	0.56	0.58	0.63	0.71	0.63
Mean BAI (cm^2^)	2.5	3.0	2.2	9.3	8.8
Chronology length (calendar yrs)	1928–2005	1925–2005	1929–2005	1921–2005	1926–2005

All characteristics, except chronology length, were computed over the period of analysis spanning from 1950 to 2005. BAI, basal area increments.

**Table 2:** Summary of stand attributes per composition type (mean value and range in parentheses).

Stand composition type	Mean basal area (m^2^ ha^−1^)	Mean tree density (no. of stems ha^−1^)
Pure black spruce	45 (31–56)^a^	3731 (1800–5275)^b^
Mixed	54 (40–64)^ab^	1916 (1125–2650)^ab^
Pure trembling aspen	58 (42–73)^b^	1313 (825–2025)^a^

Pure stands were defined as containing >75% of the dominant species in relative basal area, whereas mixed stands had ≤75% of one species in relative basal area. Stems with diameter at breast height ≥5 cm were used. Different letters represent significant differences between median values (*p* < 0.05); the order of the letter marks is ascending.

### Climate-Growth Relations

**Figure 2:**
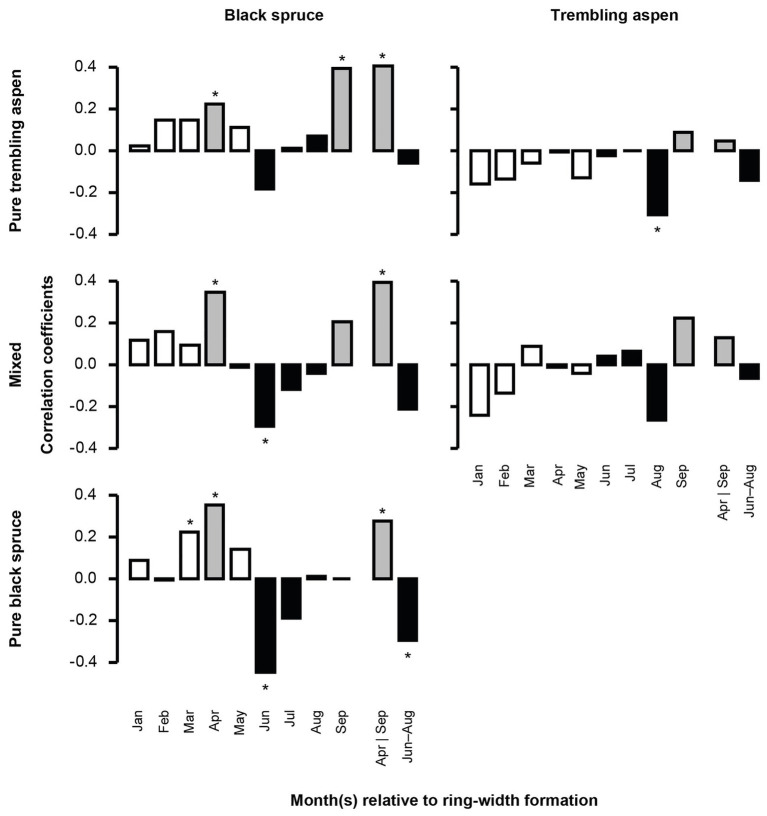
Correlation between monthly average temperature and mean basal area increment (BAI) chronologies of black spruce and trembling aspen in pure and mixed stands from 1950 to 2005. April and September, important months for growing season length, are in gray; June–August, determining summer conditions, are in black. Stars represent significant correlation coefficients (*p* < 0.05). In each analysis, a 9-month window from January to September, and the mean values for April and September, and June–August of the year of ring formation were evaluated.

Mean BAI was positively correlated with higher temperatures in the early months of spring (March–April) for black spruce regardless of stand mixture, and in September for black spruce in PTA (Figure 2; Supplementary Figures 2, 3). Combined, April and September temperatures were positively correlated with mean BAI for black spruce regardless of stand mixture, indicating that black spruce may respond positively to longer growing seasons. Compared to black spruce, trembling aspen had no significant correlations between mean BAI and April and September temperatures.

**Figure 3:**
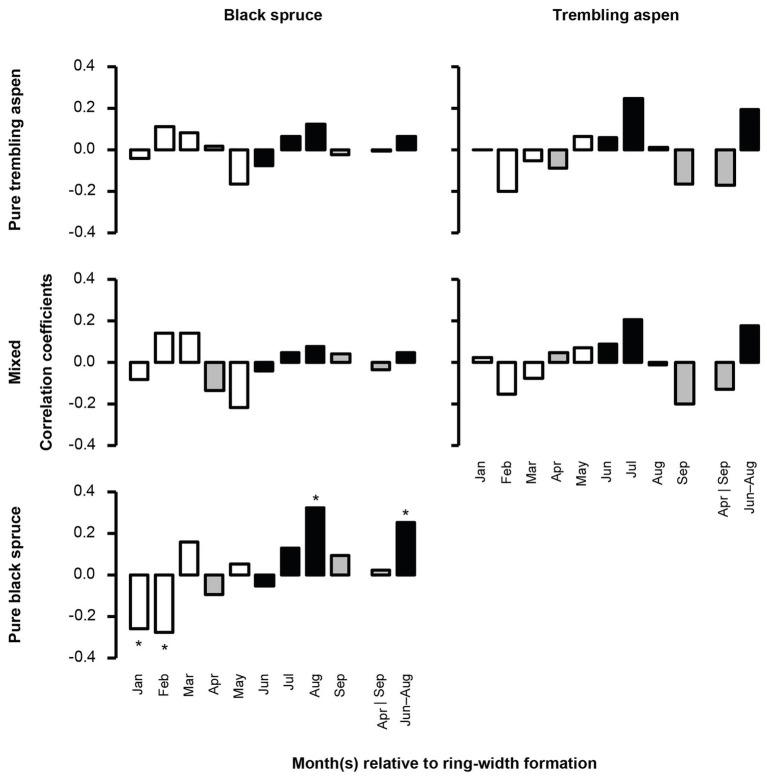
Correlation between total monthly precipitation and mean BAI chronologies of black spruce and trembling aspen in pure and mixed stands from 1950 to 2005. April and September, important months for growing season length, are in gray; June–August, determining summer conditions, are in black. Stars represent significant correlation coefficients (*p* < 0.05). In each analysis, a 9-month window from January to September, and the mean values for April and September, and June–August of the year of ring formation were evaluated.

Mean BAI was negatively correlated with higher temperatures in June and July for black spruce in pure black spruce and mixed stands, and in August for trembling aspen, suggesting that both species may be negatively impacted by summer heat stress. During winter months (January and February), increased precipitation was negatively correlated with mean BAI for black spruce in pure spruce stands (Figure 3), and for the same stands positive correlations with August precipitation were detected, indicating some hydric limitations in the late summer. For trembling aspen, no significant correlations with precipitation were detected.

### Influence of Specific Epidemic Years on Host and Non-host Species

**Figure 4:**
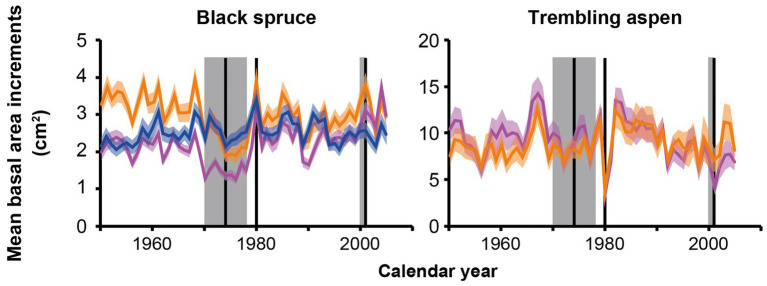
Mean BAIs for black spruce and trembling aspen from 1950 to 2005. The blue curve represents mean BAI in pure black spruce stands (PBS), the orange curves represent mean BAI in mixed stands, and the purple curves represent mean BAI in pure trembling aspen stands (PTA). The colored areas represent ±1 SEM. The gray areas and black vertical lines correspond to the spruce budworm epidemic centered around 1974, and the forest tent caterpillar (FTC) epidemics of 1980 and 2000–2001 (Bergeron et al., 2002; Ministère des Forêts, de la Faune et des Parcs, 2020c).

SBW and FTC epidemics revealed distinct impacts on growth depending on composition type (Figure 4; Supplementary Table 1). The SBW epidemic recorded in the 1970s coincided with decreases in mean BAI from 1 year to the next for black spruce in 1970, 1973, and 1974 across PBS (5th, 22nd, and 6th largest decreases, respectively), mixed stands (3rd, 7th, and 8th largest decreases, respectively), and PTA (2nd, 9th, and 14th largest decreases, respectively).

Forest tent caterpillar epidemics recorded in 1980, 2000, and 2001 coincided with decreases in mean BAI from 1 year to the next for trembling aspen across PTA (1st, 10th, and 2nd largest decreases, respectively) and mixed stands (1st, 15th, and 13th largest decreases, respectively). For black spruce in mixed and PTA, 1980 corresponded to large increases in mean BAI from 1 year to the next (3rd and 1st largest increases, respectively). This suggests that black spruce benefitted from an opening of the canopy due to FTC epidemics.

### Interactions Among Tree Growth, Species Mixture, Climate, and Insect Epidemics

The linear Bayesian models summarized the growth responses of specific mean BAI chronologies to site conditions, climate, and insect epidemics. The models explained moderate to high proportions of BAI variability (*R*^2^ = 28–67%; Supplementary Figure 4). No convergence issues in parameter selection were detected based on unimodal distributions of parameter posterior probabilities, shrinking of these probabilities relative to prior ranges, and stability of parameter values over MCMC chains (Supplementary Figures 5–13; Supplementary Table 2).

**Figure 5:**
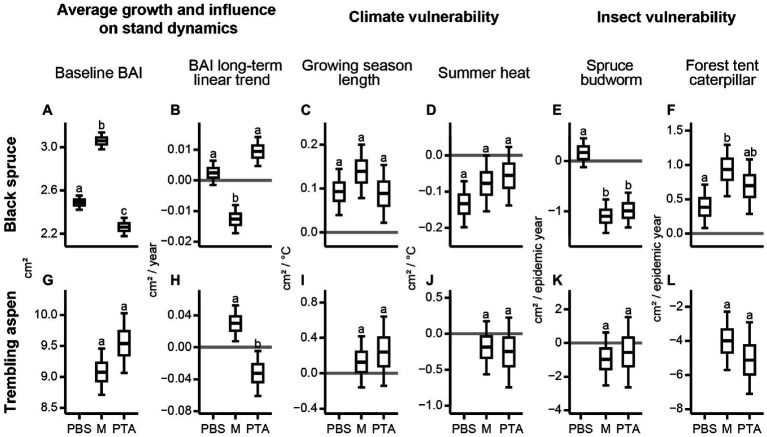
Posterior distributions for parameters of the linear Bayesian models used to explain mean BAI chronologies for specific composition type as a function of climate and occurrence of insect epidemics. Boxplots compare the parameters for black spruce and trembling aspen in PBS, mixed stands (M), and PTA. Different letters represent posterior distributions with <10% overlap. We show the posterior distributions for (**A,G**) baseline BAI, (**B,H**) BAI long-term linear trend, (**C,I**) growing season length, (**D,J**) summer heat, (**E,K**) spruce budworm, and (**F,L**) forest tent caterpillar according to species and composition type.

Most significant differences across posterior distributions were for black spruce in its three stand composition types. Black spruce average growth, indicated by baseline mean BAI, was significantly greater in mixed stands than in pure stands, and significantly greater in PBS than in PTA (Figure 5A). Mean BAI long term-linear trends were negative for black spruce in mixed stands and positive for PTA (Figure 5B). Regardless of composition type, black spruce responded positively to longer growing seasons and negatively to higher summer heat stress. Although differences among stand composition types were not significant, spruce in mixed stands benefitted most from longer growing seasons (Figure 5C) and was least vulnerable to summer heat stress (Figure 5D). Black spruce in mixed and pure aspen stands was more vulnerable to SBW epidemics (Figure 5E); however, these spruce trees benefitted from the negative impact of FTC epidemics on aspen (Figure 5F).

Compared to black spruce, trembling aspen did not reveal significant differences across its two stand composition types (Figures 5G,I–L), except for the mean BAI long-term linear trend, which was significantly greater in mixed stands (Figure 5H). Both aspen chronologies were negatively affected by the FTC epidemics, which halved mean BAI during epidemic years (Figure 5L).

## Discussion

We aimed to assess how climate and insect epidemics impact the growth of boreal tree species in mixed and pure stands of eastern Canada because species mixture may be a potential strategy to promote resilience of boreal forests in the context of global change by increasing tree growth (Hisano et al., 2019), reducing tree climate vulnerability to extreme events, and attenuating the impact of insect epidemics (Drobyshev et al., 2013). Here, we verify if some of these potential beneficial effects are visible in the mean BAI values of key boreal tree species in mixed and pure stands of eastern Canada: black spruce and trembling aspen. The results of our study are summarized in Table 3, and Figure 5, which is used to organize the following discussion.

**Table 3:** Summary of the effects of species mixture on responses of black spruce and trembling aspen based on the results of the linear Bayesian models (Figure 5).

	Responses of		
	Black spruce in mixed vs. pure black spruce stands	Black spruce in pure trembling aspen vs. pure black spruce stands	Trembling aspen in mixed vs. pure trembling aspen stands
**Average growth and influence on stand dynamics**
Baseline BAI	Higher^*^	Lower^*^	Lower^][^
BAI long-term linear trend	Lower^*^	Higher^][^	Higher^*^
**Climate vulnerability**
Growing season length	Positive response^][^	Null	Null
Summer heat	Lower vulnerability^][^	Lower vulnerability^][^	Null
**Insect vulnerability**
Insect epidemic (SBW for spruce; FTC for aspen)	Higher vulnerability^*^	Higher vulnerability^*^	Null
Insect epidemic on the companion species	Positive response^*^	Positive response^][^	Null

For example, we compared the posterior distributions of baseline mean BAIs among mixed and pure stands to determine the result (i.e., higher or lower baseline mean BAI values). We identified with ^*^responses that had overlaps between posterior distributions <10%, and with ^][^responses with non-overlapping interquartile ranges between posterior distributions. SBW, spruce budworm; FTC, forest tent caterpillar.

### Average vs. Long-Term Growth as Function of Composition Type

Our study provided evidence that trees in mixed stands can yield high mean BAI values and be less vulnerable to predicted warming in the region due to climate change. Black spruce trees in mixed stands had high baseline mean BAI relative to spruce in pure stands (Figure 5A), but their long-term growth trend was impacted by the SBW epidemic in the 1970s, which coincided with several years of above average summer drought within the region (Environment Canada, 2020b; Figure 5B). Higher average growth of black spruce in mixed stands with aspen may be due to lower stand densities than in PBS, although mixed stands had higher basal areas (Table 3), and due to niche partitioning and type of leaf litter (Cavard et al., 2011). Black spruce in mixed stands may exploit a complementary crown packaging with trembling aspen (Burns and Honkala, 1990a,b; Cavard et al., 2011), a partitioning of the soil profile between the different root systems (Mekontchou et al., 2020), and higher nutrient availability due to aspen litterfall, which is less acidic and decomposes faster than black spruce needle litter (Légaré et al., 2005; Laganière et al., 2010). These beneficial effects were reduced following the SBW epidemic and dry conditions during the 1970s, resulting in a negative mean BAI long-term linear trend for spruce in mixed stands (Figures 4, 5B) and in a positive trend for aspen in the same stands (Figure 5H), although aspen showed a growth decline after the mid-1980s (Figure 4; Supplementary Figure 4). The aspen growth decline after the mid-1980s was more evident in pure stands likely because intraspecific competition for aspen in pure stands increased over time as these trees grew to large sizes more rapidly than in mixed stands (Figure 5G). Consequently, spruce trees in pure aspen stands showed a growth suppression until the mid-1980s and a suppression release afterward, resulting in a positive mean BAI long-term linear trend (Figures 4, 5B).

### Climate-Growth Relations as a Function of Composition Type

We found black spruce was slightly less vulnerable to climate warming in mixed stands (Figures 5C,D). Although longer growing seasons and cooler summers benefitted the growth of black spruce regardless of stand mixture, black spruce in mixed stands responded more positively to longer growing seasons and was less vulnerable to summer heat stress. With warmer springs that hasten snowmelt, growth can start earlier for black spruce (Rossi et al., 2011). Growing seasons can also end later with warmer autumns, which favor the growth of black spruce especially in PTA and mixed stands. Black spruce endures less competition for water, light, and nutrients in mixed stands as aspen loses its foliage at the end of dry summers and in early fall (Constabel and Lieffers, 1996). During summer, higher temperatures lead to higher heat stress and increase moisture evaporation from shallow soils in which spruce roots grow (Way et al., 2013). These processes were attenuated in mixed stands because of niche partitioning and potential hydraulic lift from deeper rooted trembling aspen. We also found that increased precipitation as snow during winter months decreases the growth of black spruce in pure stands only. Dense PBS produce more shading, and snowmelt is delayed, especially when snow accumulation is high during cold winters. This process likely delays the beginning of the growing season for black spruce in pure spruce stands.

Compared to black spruce, trembling aspen revealed less sensitivity to climate and stand mixture (Figures 5I,J). The weak responses to climate during the year of ring formation for trembling aspen, are consistent with some other studies analyzing climate-growth relations in eastern Canadian boreal forests (e.g., Huang et al., 2010; D’Orangeville et al., 2018a), but contrast with Drobyshev et al. (2013) who found that trembling aspen responded positively to warmer and drier climate within the region.

### Vulnerability to Insect Epidemics as a Function of Composition Type

Our study highlights significant interactions of species mixture with forest responses to insect epidemics. Although black spruce is a potential host for SBW (Blais, 1957), we found spruce in mixed stands and pure aspen stands showed a greater decrease in mean BAI and thus higher vulnerability to the 1970s SBW epidemic than in pure spruce stands (Figure 5E). The presence of aspen as a non-host species did not decrease the vulnerability to SBW of the host species as found in other studies within eastern Canada (e.g., Bergeron et al., 1995; Cappuccino et al., 1998; Campbell et al., 2008). However, these studies focused their analyses on balsam fir [*Abies balsamea* (L.) Mill.], the preferred diet of SBW (Hennigar et al., 2008). Two potential explanations may be proposed for this unexpected result. First, within the study area, Nagati et al. (2019) found a spatial association between balsam fir and trembling aspen linked to higher litter nutrient availability, which supports mycorrhizal communities associated with balsam fir. In our plots, we noticed the presence of balsam fir regeneration mainly in aspen dominated stands and we hypothesized that this presence may have contributed to the concentration of SBW on the nearby available spruce trees. Second, studies reporting a positive effect of species mixture on the attenuation of SBW impact analyzed species composition at the landscape/regional level (e.g., Campbell et al., 2008). In the region encompassing our study area, black spruce stands tend to dominate the landscape. The effect of species mixture in this condition may be less relevant than in a patchier landscape limiting SBW diffusion.

Conversely, the FTC epidemics in 1980 and 2000–2001 affected aspen trees severely, with a similar magnitude across mixture categories (Figure 5L), while black spruce in mixed and pure aspen stands had simultaneous and significant positive responses in growth, likely linked to higher light and water availability (Figure 5F). This implies that stand mixture may contribute to the stabilization of biomass increments over time at the ecosystem level, even when one species is affected by growth depletions in particular years. Similar findings on tree biomass stabilization in mixed stands of temperate and boreal forests of eastern Canada were reported by Aussenac et al. (2016). Differing growth responses between species associated with climatic fluctuations and insect epidemics could compensate thereby attenuating negative impacts on stand biomass accumulation (Aussenac et al., 2016).

## Conclusion

In boreal forests of eastern Canada, stand species mixture offered several benefits against vulnerability to climate change and insect epidemics. Specifically, we found that black spruce in mixed stands had on average higher tree biomass accumulation than in pure stands; although, the long-term growth trend in mixed stands was negative due to stand dynamics and the 1970s SBW epidemic. In mixed stands, black spruce was less vulnerable to summer heat stress than in pure stands and benefitted from longer growing seasons. In contrast, trembling aspen was overall less sensitive to climate than black spruce, regardless of stand mixture. Within mixed relative to pure stands, both spruce and aspen did not show any reduction of vulnerability to insect epidemics as a host species, but spruce responded positively to FTC epidemics affecting aspen.

Our findings revealed the complex interactions of tree species in mixed stands and provide partial support toward forest management strategies that promote species mixture to decrease forest vulnerability to climate change and insect epidemics. For example, tree species responses to insect epidemics seemed to be affected by multiple interactions at stand and study area scales masking the effect of species mixture; although, contrasting growth responses between host and non-host tree species could stabilize carbon accumulation of mixed stands over time. Clearly, further research is warranted to disentangle the strength and direction of these interactions for boreal tree species in different mixtures and potentially other environmental conditions.

## Data Availability Statement

The original contributions presented in the study are included in the article/Supplementary Material, further inquiries can be directed to the corresponding author.

## Author Contributions

XC: data curation. RC and FG: formal analysis, methodology, visualization, and writing— original draft. RC, FG, and YB: investigation. YB and FG: resources and supervision. RC, FG, YB, XC, PG, and HM: writing – review and editing. All authors contributed to the article and approved the submitted version.

### Conflict of Interest

The authors declare that the research was conducted in the absence of any commercial or financial relationships that could be construed as a potential conflict of interest.
