# Supplementary material for: Role of Mixed-Species Stands in Attenuating the Vulnerability of Boreal Forests to Climate Change and Insect Epidemics
Source: Front Plant Sci. 2021 Apr 29;12:658880. doi: 10.3389/fpls.2021.658880 (PMC8117013; doi:10.3389/fpls.2021.658880)
Supplement: Supplementary file 1 [file Data_Sheet_1.PDF]

Supplementary Material

1 Supplementary Figures and Tables

1.1 Supplementary Figures

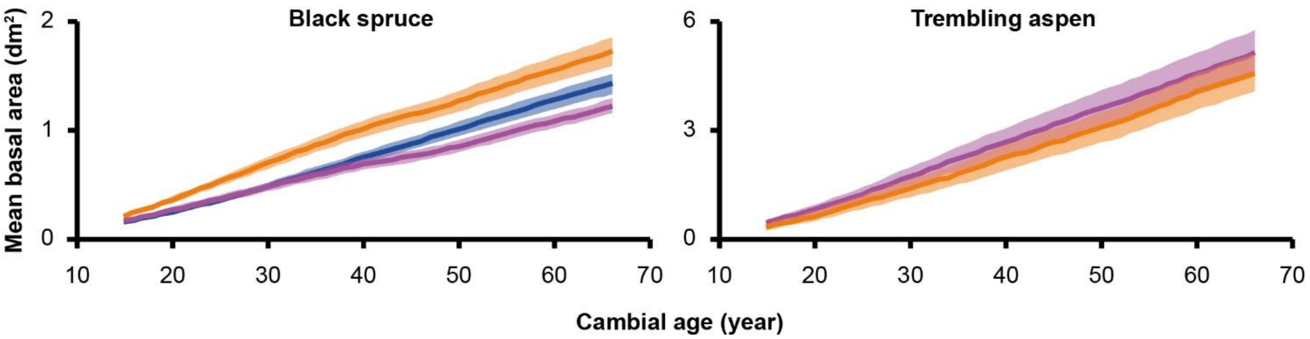

Supplementary Figure 1. Mean basal area of black spruce and trembling aspen according to cambial age in pure black spruce stands (blue curve), mixed stands (orange curves), or pure trembling aspen stands (purple curves). The colored areas represent ±1 standard errors of the mean.

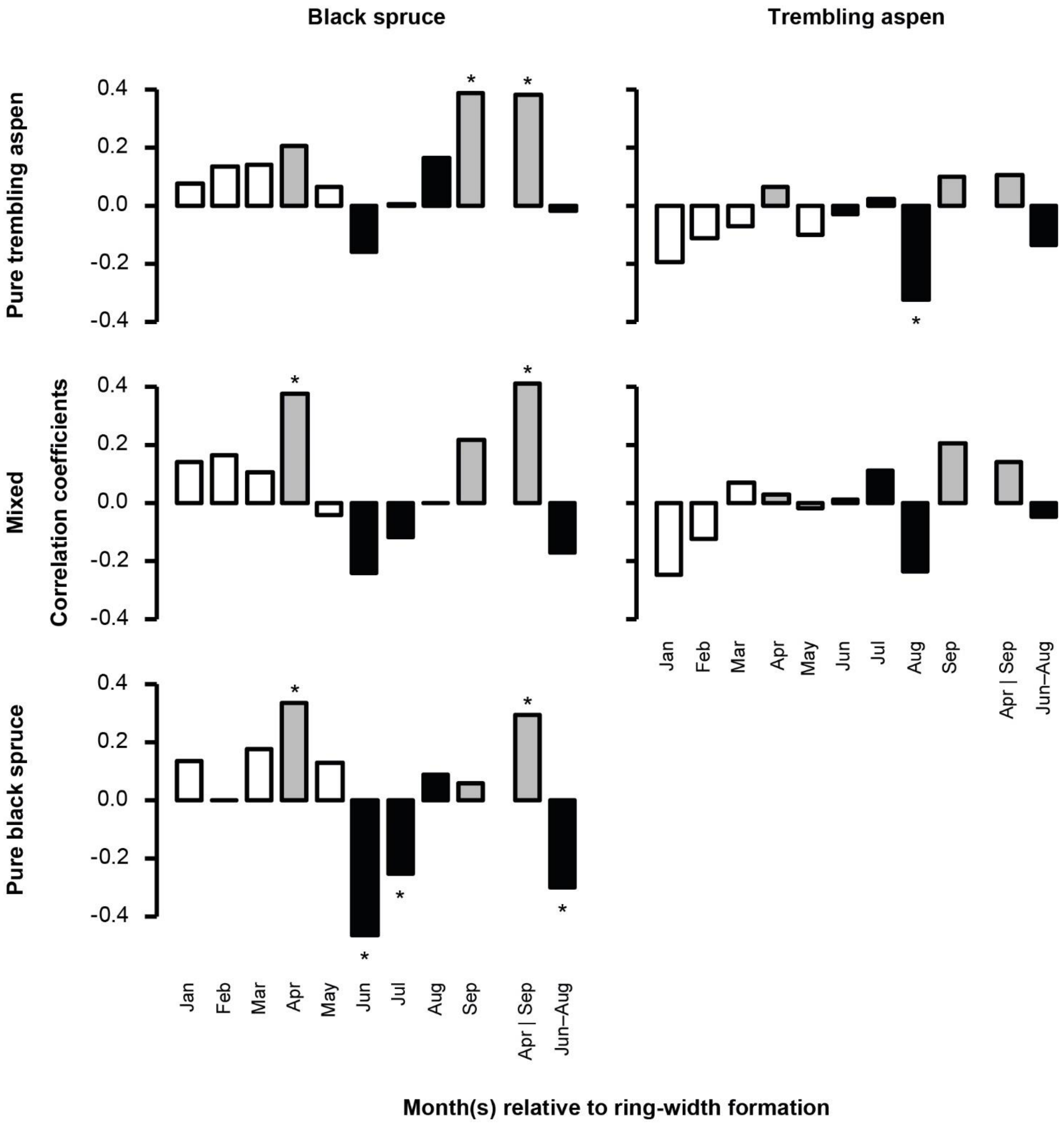

Supplementary Figure 2. Correlation between monthly minimum temperature and basal area increment chronologies of black spruce and trembling aspen in pure and mixed stands from 1950–2005. April and September, important months for growing season length, are in gray; June–August, determining summer conditions, are in black. Stars represent significant correlation coefficients ($p < 0.05$). In each analysis, a 9-month window from January to September, and the mean values for April and September, and June–August of the year of ring formation were evaluated.

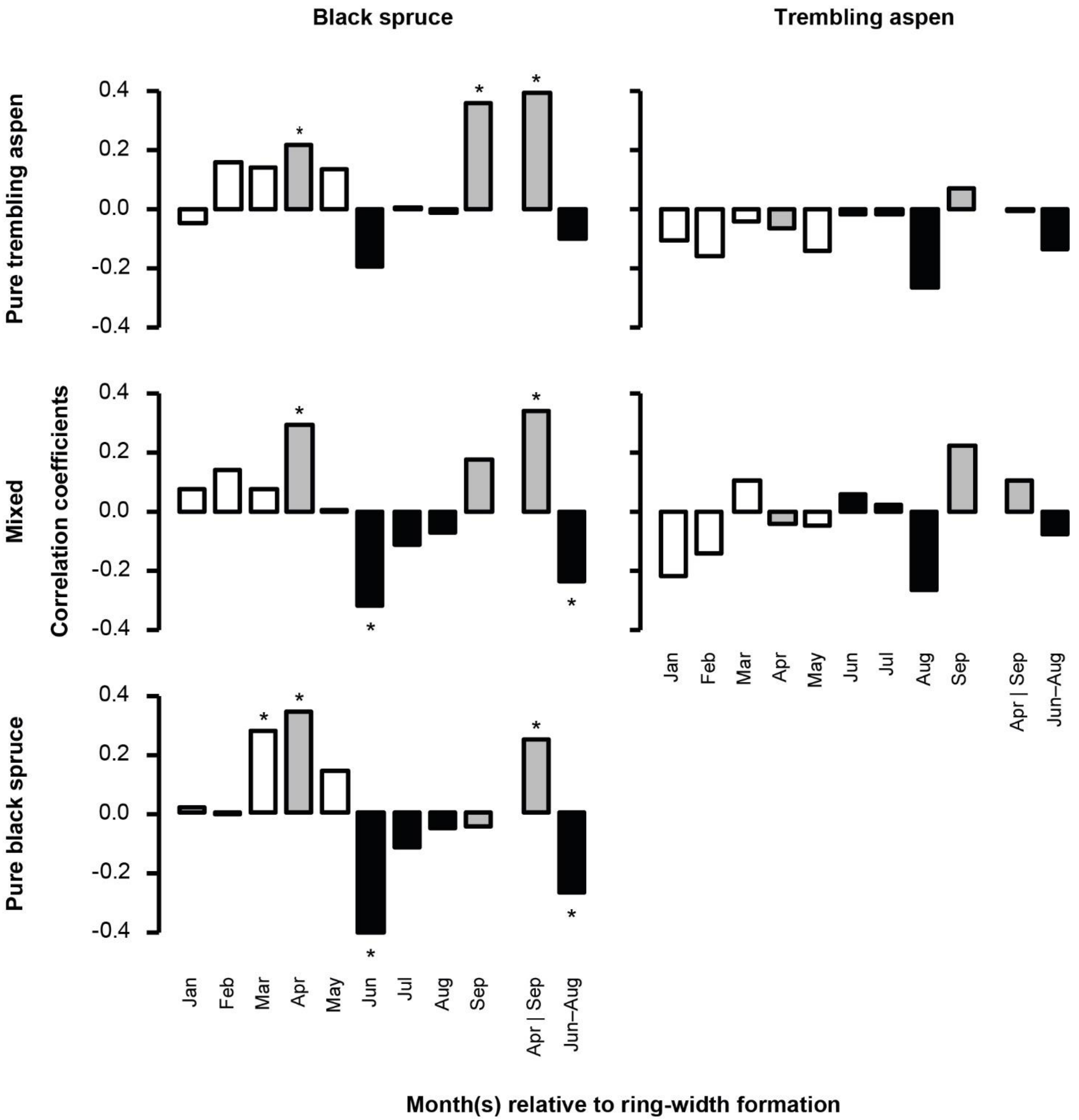

Supplementary Figure 3. Correlation between monthly maximum temperature and basal area increment chronologies of black spruce and trembling aspen in pure and mixed stands from 1950–2005. April and September, important months for growing season length, are in gray; June–August, determining summer conditions, are in black. Stars represent significant correlation coefficients ($p < 0.05$). In each analysis, a 9-month window from January to September, and the mean values for April and September, and June–August of the year of ring formation were evaluated.

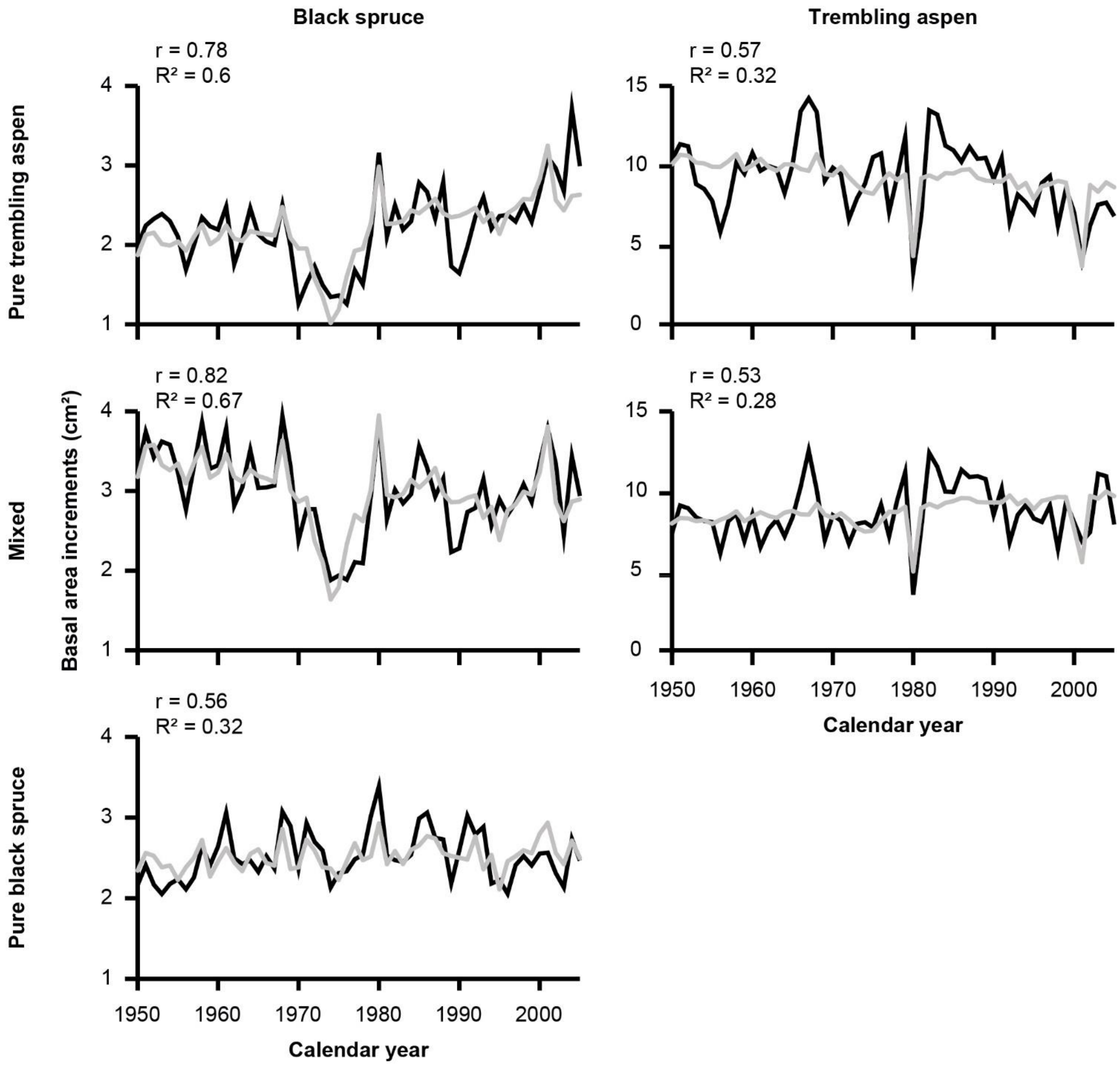

Supplementary Figure 4. Observed versus predicted basal area increment time series (black and gray curves, respectively) of black spruce and trembling aspen in pure and mixed stands. Predicted values were obtained with multiple linear Bayesian models.

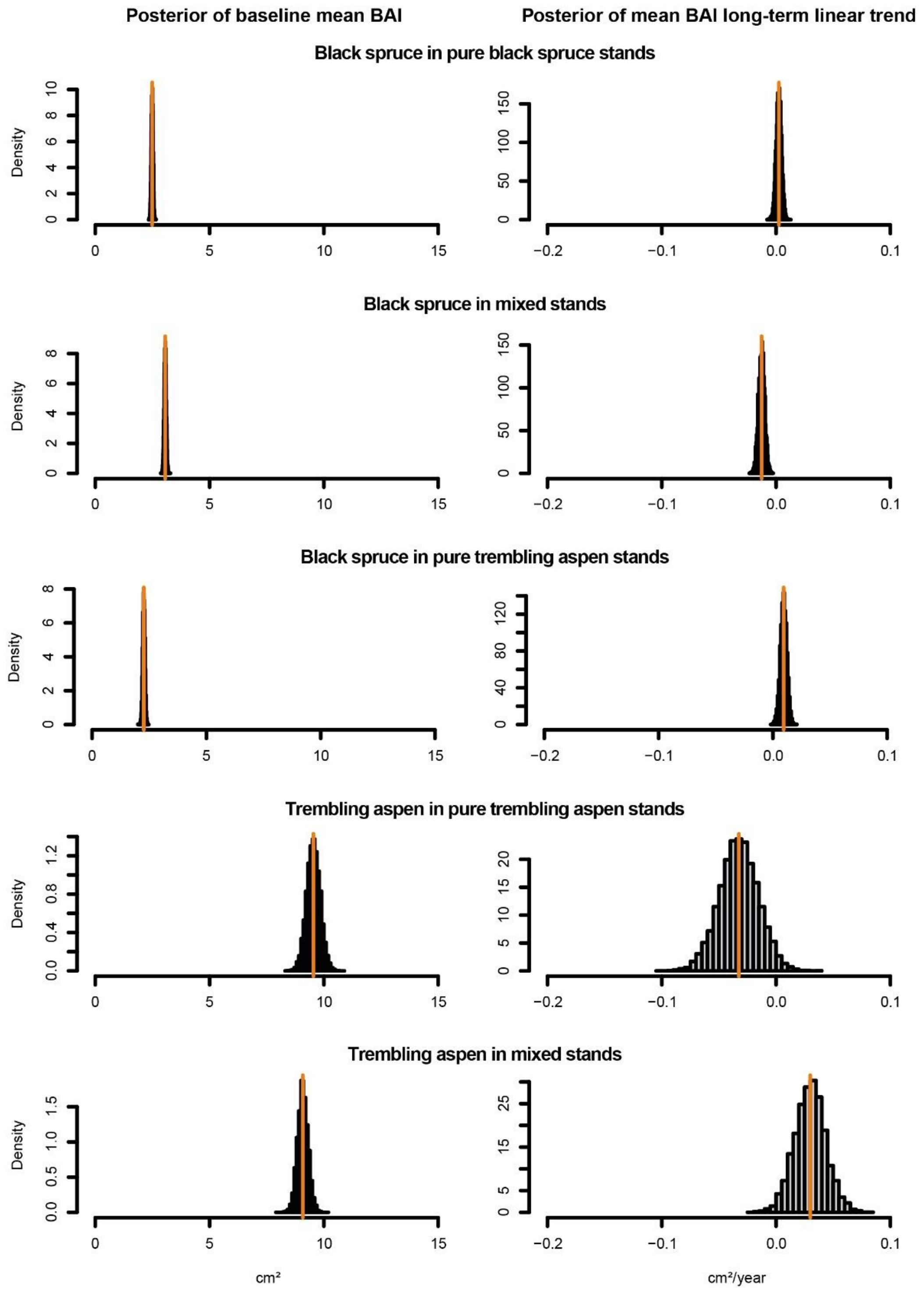

Supplementary Figure 5. Posterior distributions for parameters of the Bayesian models (baseline mean basal area increments and mean BAI long-term linear trend) with chronologies for black spruce and trembling aspen in mixed and pure stands (orange line = mean).

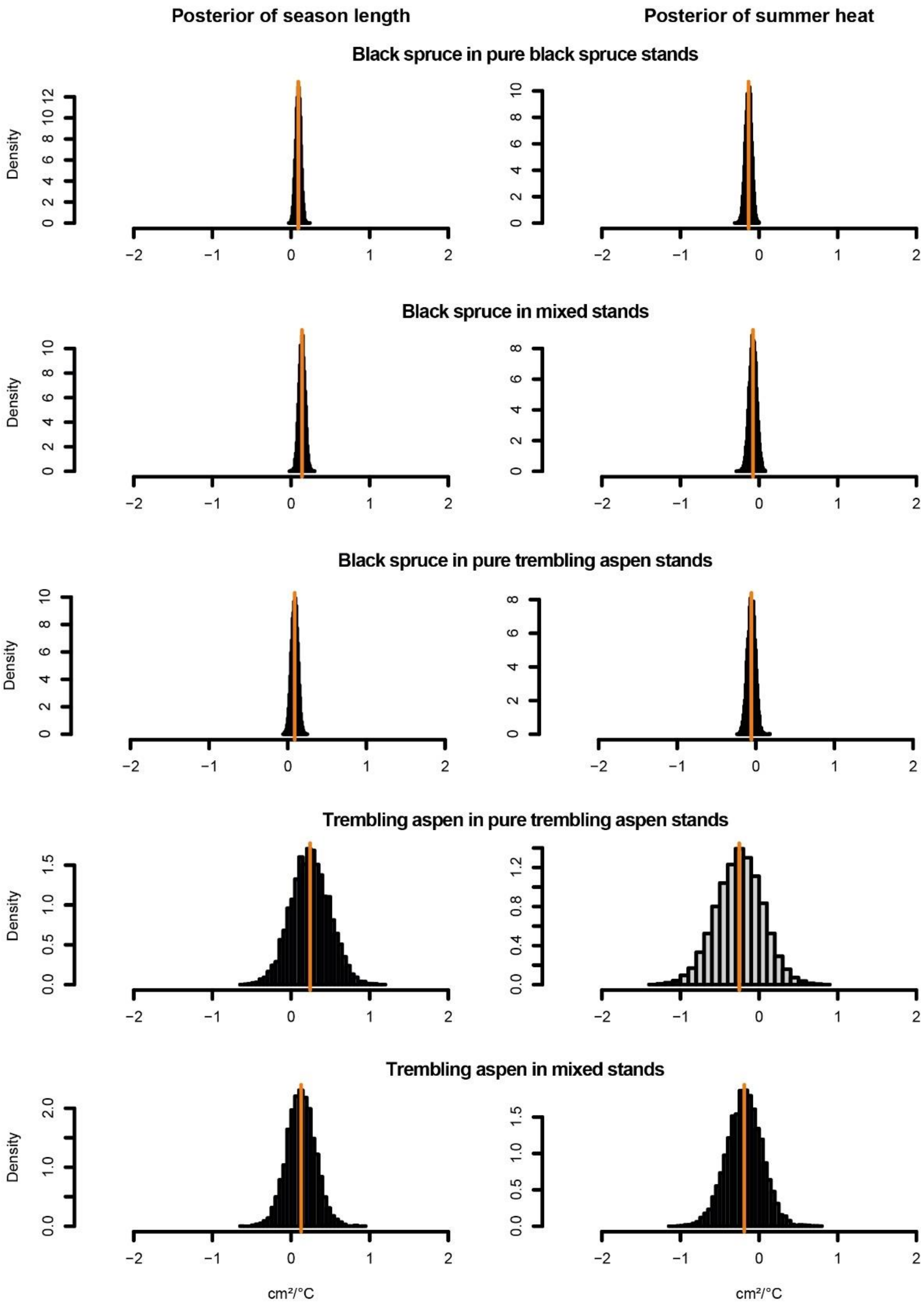

Supplementary Figure 6. Posterior distributions for parameters of the Bayesian models (growing season length and summer heat stress) with chronologies for black spruce and trembling aspen in mixed and pure stands (orange line = mean).

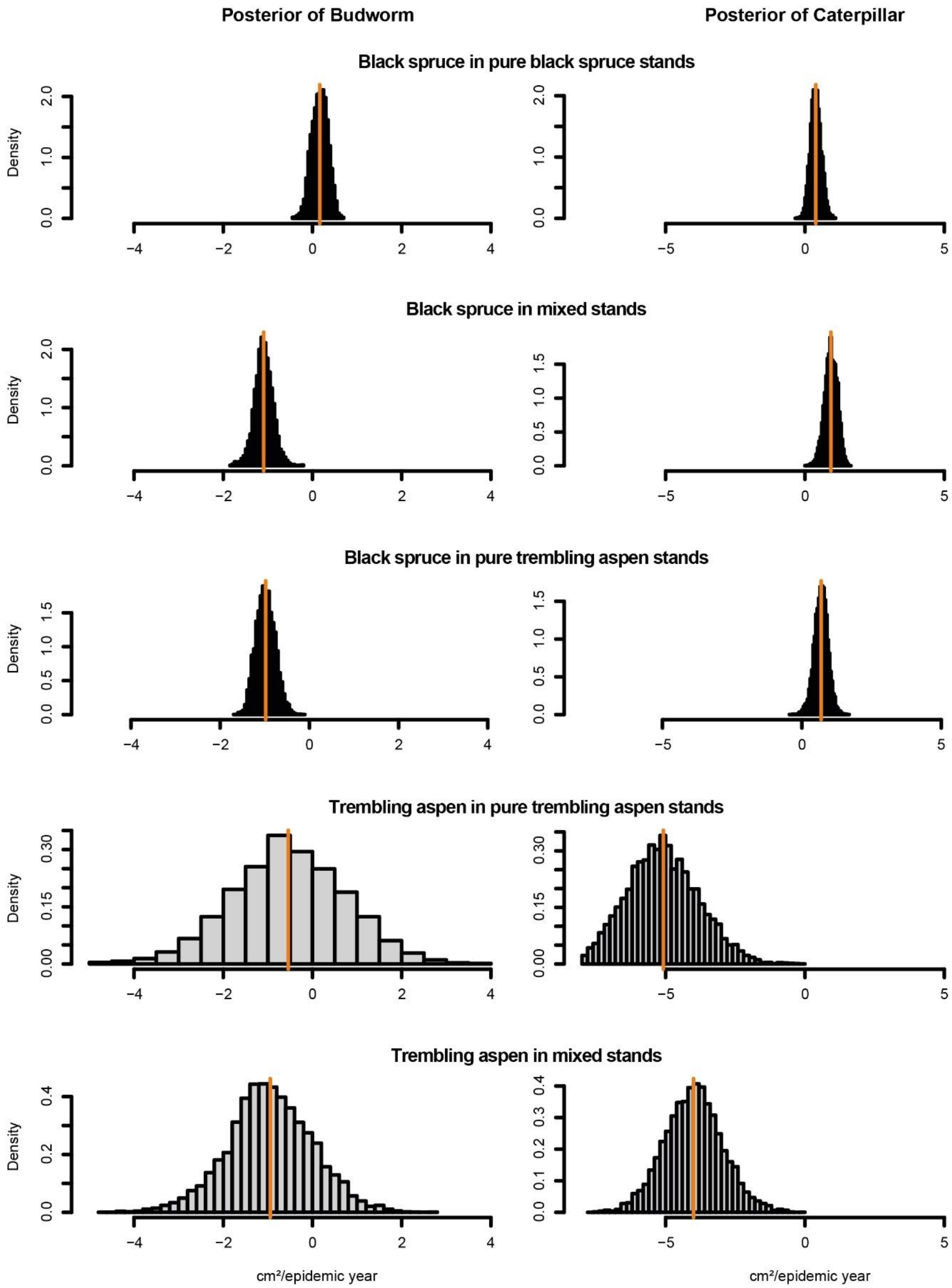

Supplementary Figure 7. Posterior distributions for parameters of the Bayesian models (spruce budworm and forest tent caterpillar epidemics) with chronologies for black spruce and trembling aspen in mixed and pure stands (orange line = mean).

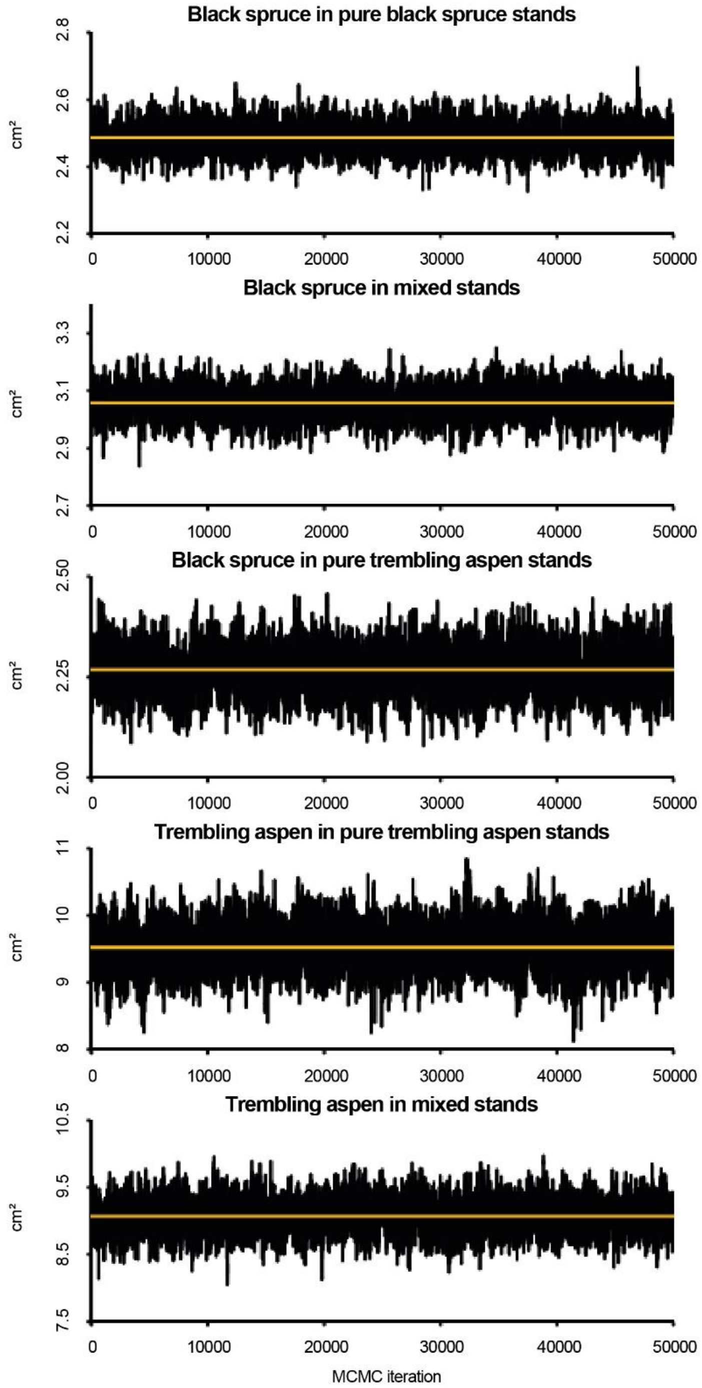

Supplementary Figure 8. Baseline mean basal area increment chain values for parameters over retained Markov Chain Monte Carlo (MCMC) iterations with chronologies for black spruce and trembling aspen in mixed and pure stands (orange line = mean).

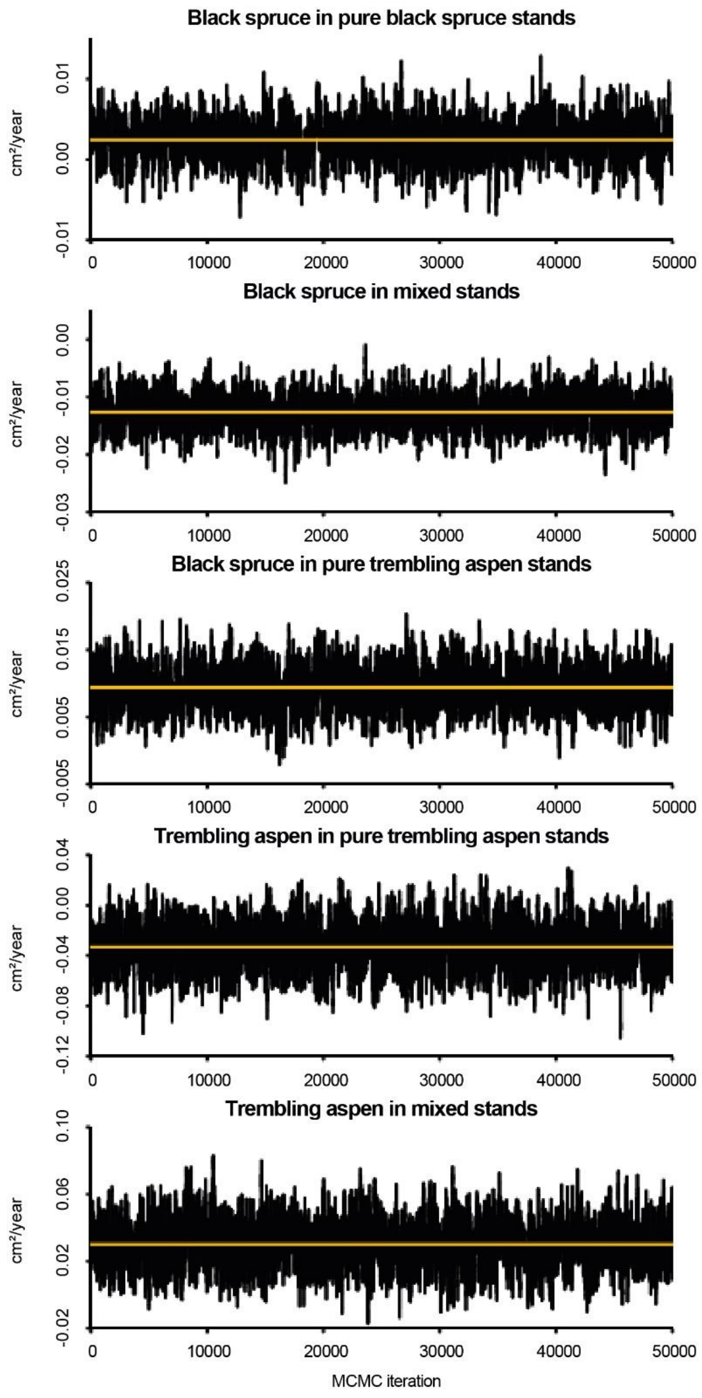

Supplementary Figure 9. Mean basal area increment long-term linear trend chain values for parameters over retained Markov Chain Monte Carlo (MCMC) iterations with chronologies for black spruce and trembling aspen in mixed and pure stands (orange line = mean).

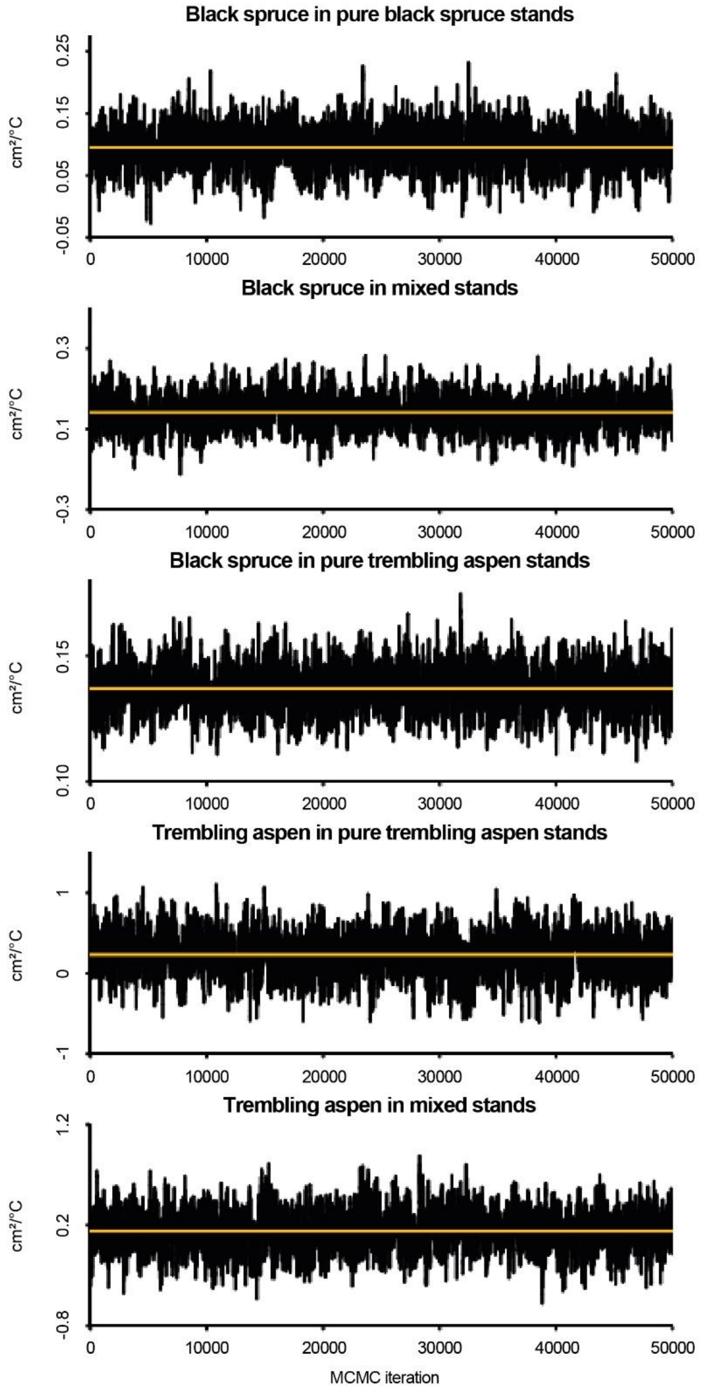

Supplementary Figure 10. Growing season length chain values for parameters over retained Markov Chain Monte Carlo (MCMC) iterations with chronologies for black spruce and trembling aspen in mixed and pure stands (orange line = mean).

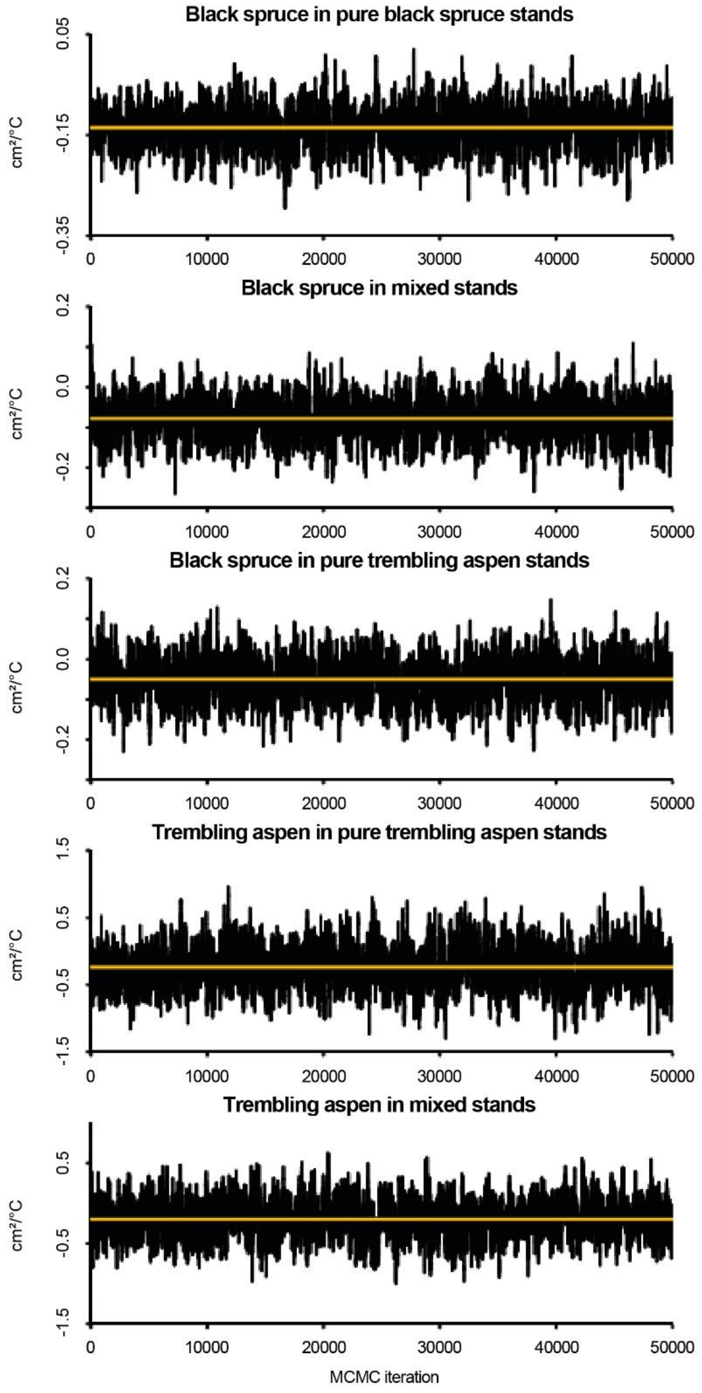

Supplementary Figure 11. Summer heat stress chain values for parameters over retained Markov Chain Monte Carlo (MCMC) iterations with chronologies for black spruce and trembling aspen in mixed and pure stands (orange line = mean).

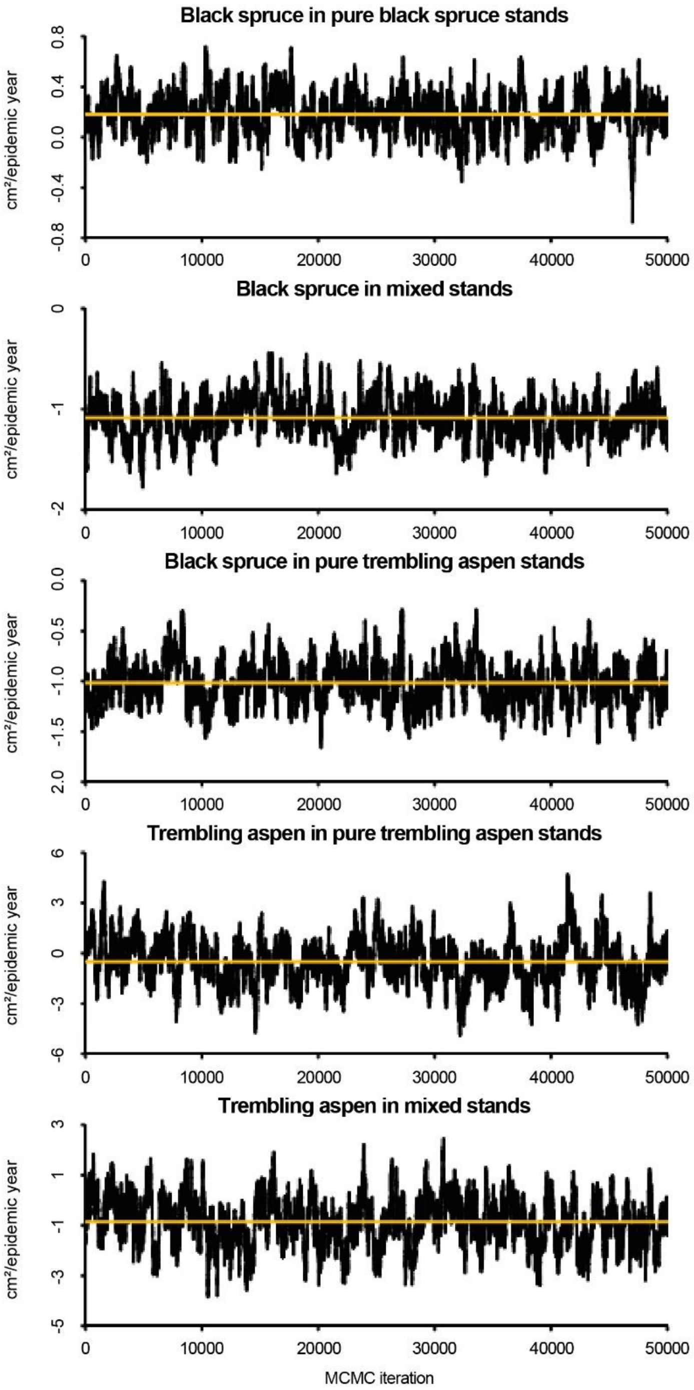

Supplementary Figure 12. Spruce budworm epidemic chain values for parameters over retained Markov Chain Monte Carlo (MCMC) iterations with chronologies for black spruce and trembling aspen in mixed and pure stands (orange line = mean).

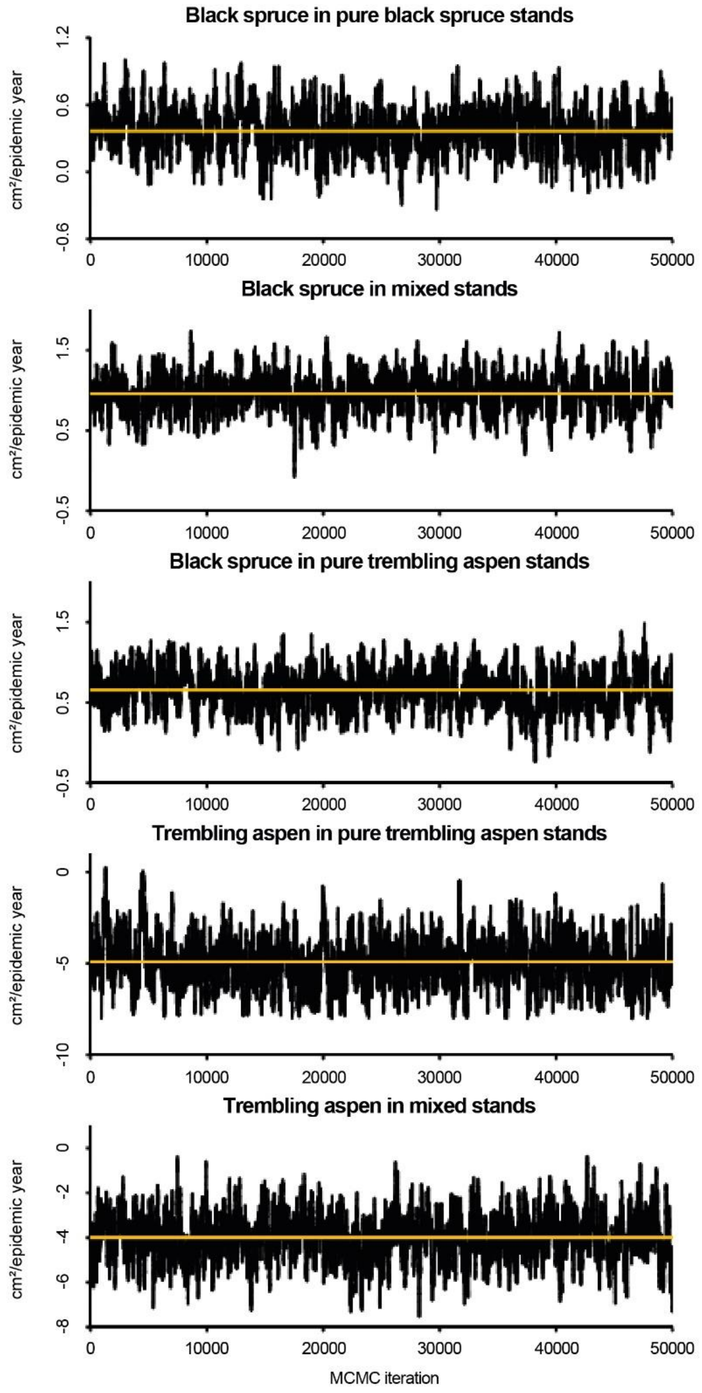

Supplementary Figure 13. Forest tent caterpillar epidemic chain values for parameters over retained Markov Chain Monte Carlo (MCMC) iterations with chronologies for black spruce and trembling aspen in mixed and pure stands (orange line = mean).

1.2 Supplementary Tables

Supplementary Table 1. Ranked years of increases and decreases in mean basal area increments from one year to the next (i.e., first-differenced chronologies) for black spruce and trembling aspen in pure and mixed stands from 1950–2005. Blue areas represent the spruce budworm epidemic centered around 1974 (±4 yrs), whereas gray areas represent forest tent caterpillar epidemics of 1980 and 2000–2001.

		Black spruce			Trembling aspen	
	Rank	Pure black spruce	Mixed	Pure trembling aspen	Pure trembling aspen	Mixed
Increases	1	1968	1979	1980	1981	1981
	2	2004	2004	1979	1982	1982
	3	1971	1980	2004	2002	2003
	4	1979	1968	1977	1999	1999
	5	1990	1985	1968	1958	1957
	6	1985	1991	1985	1966	1978
	7	1997	1957	1991	1978	1993
	8	1958	2000	1971	1957	1960
	9	1991	1958	1988	1993	1951
	10	1961	1971	1992	1996	1966
Decreases	1	1981	1981	1989	1980	1980
	2	1994	1989	1970	2001	1992
	3	1989	1970	1981	1992	1969
	4	1962	2003	1962	1977	1998
	5	1970	1962	1969	1998	2005
	6	1974	1994	1956	1969	1956
	7	1987	1973	2005	1972	1961
	8	1952	1974	1994	1956	1977
	9	2002	1969	1973	1953	1990
	10	2005	1959	1965	2000	1959

Supplementary Table 2. Prior ranges for the parameters of the models explaining BAI. $BAI_{t,y}$ = $\alpha\_Baseline_y$ + $Trend_t$ α_Tr_y + $SeasonLength_t$ α_SL_y + $SummerHeat_t$ α_SH_y + $Budworm_t$ α_SB_y + $Caterpillar_t$ α_TC_y.

Parameter	Low boundary	High boundary
α_Baseline (cm^2)	0	20
α_Tr (cm^2/year)	-0.2	0.2
α_SL (cm^2/°C)	-3	3
α_SH (cm^2/°C)	-3	3
α_SB (cm^2/epidemic year)	-5	5
α_TC (cm^2/epidemic year)	-8	8
σ (cm^2)	0	10
